# Supplementary material for: Gene expression profile of the developing endosperm in durum wheat provides insight into starch biosynthesis
Source: BMC Plant Biol. 2023 Jul 18;23:363. doi: 10.1186/s12870-023-04369-7 (PMC10353290; doi:10.1186/s12870-023-04369-7)
Supplement: Supplementary file 7 — (Figures) [file 12870_2023_4369_MOESM7_ESM.pdf]

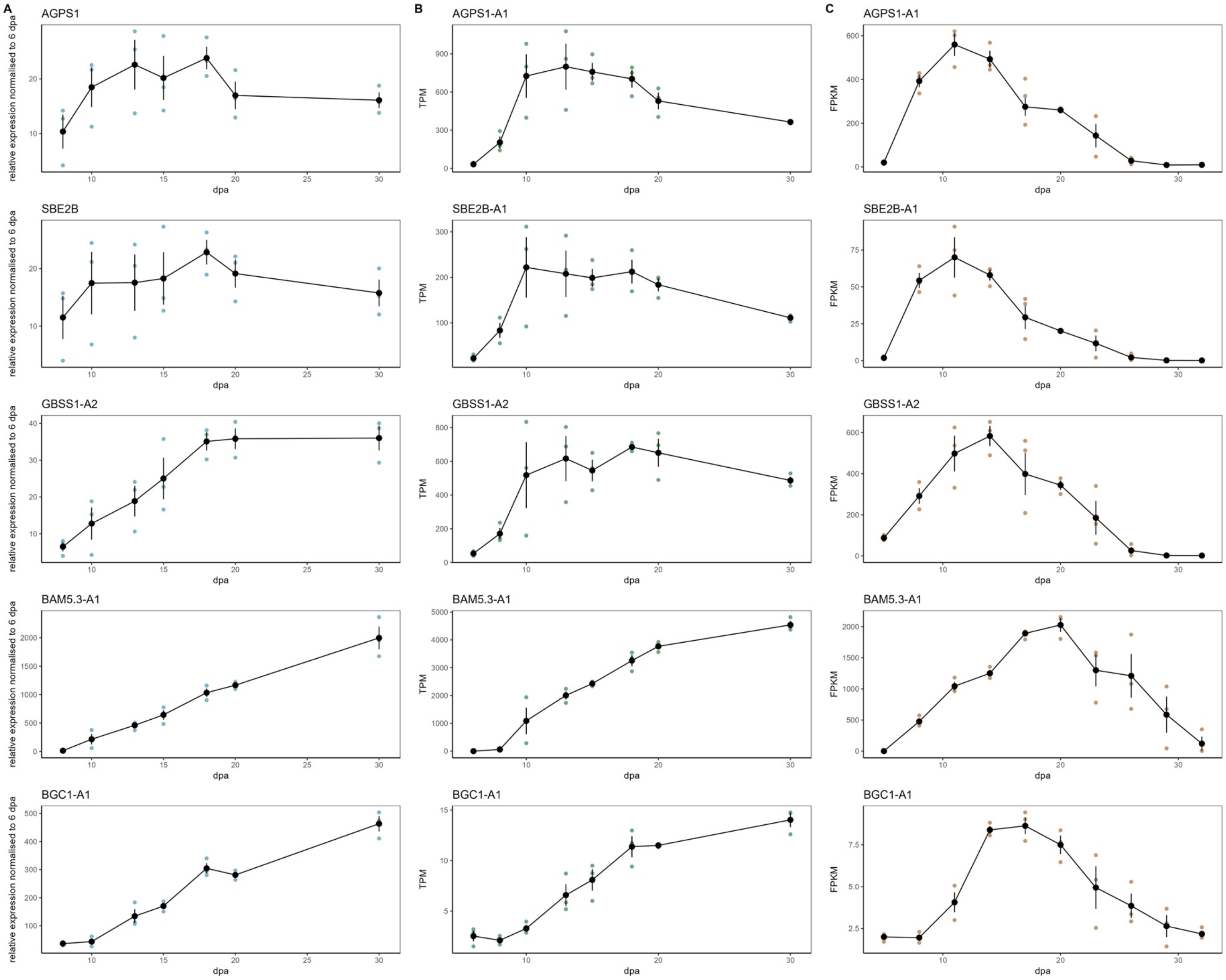
A
AGPS1
SBE2B
GBSS1-A2
BAM5.3-A1
BGC1-A1
relative expression normalised to 6 dpa
dpa
B
AGPS1-A1
SBE2B-A1
GBSS1-A2
BAM5.3-A1
BGC1-A1
TPM
dpa
C
AGPS1-A1
SBE2B-A1
GBSS1-A2
BAM5.3-A1
BGC1-A1
FPKM
dpa

Supplemental Figure 1. qRT-PCR quantification of selected starch-related genes show expression patterns consistent with the RNAseq. A) Relative expression of five starch metabolism genes in durum wheat over endosperm development, measured using qRT-PCR. The relative expression was calculated using the ΔΔCt method, normalised to ATG8d [76] and the average Ct value of the 6 dpa time point measurements. Primers for AGPS1 and SBE2b were obtained from [76] and were not homeolog-specific. **B)** Expression patterns of starch metabolism genes in durum wheat, expressed in TPM, from our current study. **C)** Expression patterns of starch metabolism genes in hexaploid bread wheat, expressed in FPKM from Gu et al. [22].

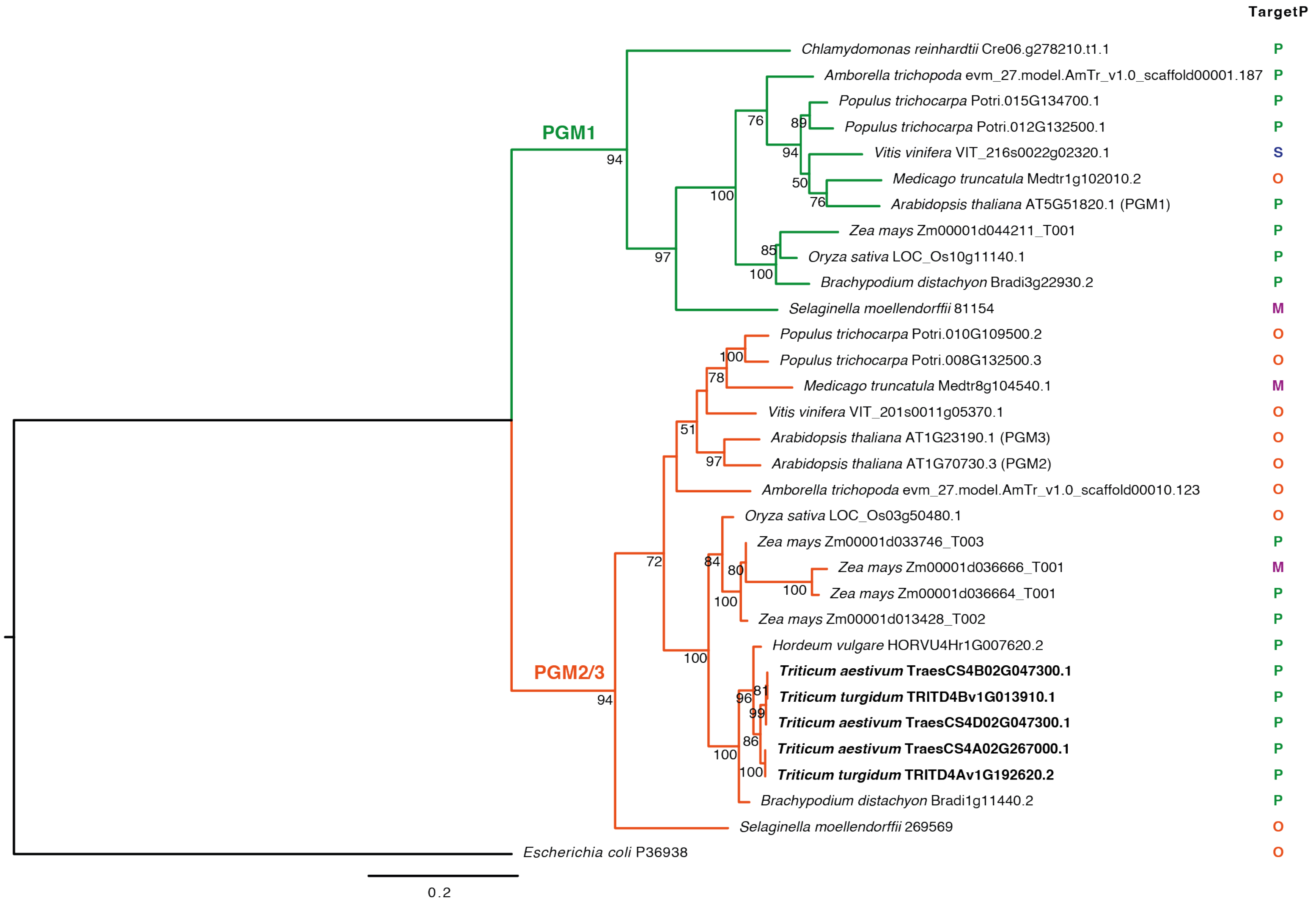

Supplemental Figure 2: Phylogenetic tree of PGM isoforms. Sequences of PGM isoforms were obtained from Phytozome v13 (Goodstein et al., 2012; doi: 10.1093/nar/gkr944). Sequences were aligned using MAFFT (Rozewicki et al., 2019; doi: 10.1093/nar/gkz342) and a maximum likelihood tree was constructed using RAxML (Stamatakis 2014; doi: 10.1093/bioinformatics/btu033) with 1,000 bootstrap replicates. Bootstrap values greater than 50 are shown next to the node. Branch lengths represent the number of substitutions per site, indicated by the scale bar. TargetP (Emanuelsson et al., 2007; doi: 10.1038/nprot.2007.131) was used to predict the subcellular targeting of each isoform, either as plastidial (P), mitochondrial (M), secreted (S) or other (O).
